# Supplementary material for: Determining the effects of temperature on the evolution of bacterial tRNA pools
Source: bioRxiv. 2023 Oct 9:2023.09.26.559538. Preprint. [Version 2] doi: 10.1101/2023.09.26.559538 (PMC10592612; doi:10.1101/2023.09.26.559538)
Supplement: Supplement 1 [file media-1.pdf]

Determining the effects of temperature on the evolution of bacterial tRNA pools

Vatsal Jain[1], Alexander L. Cope[2, 3, *]

[1]Biotechnology High School, Freehold, New Jersey; [2]Department of Genetics, Rutgers University, Piscataway, New Jersey; [3]Human Genetics Institute of New Jersey, Rutgers University, Piscataway, New Jersey; [4]Robert Wood Johnson Medical School, Rutgers University, Piscataway, New Jersey; *Corresponding author: alexander.cope@rutgers.edu

Supplemental Tables

Phylogenetic Regression	Model	AIC	ΔAIC (relative to best)
GC vs. tRNA Diversity	BM	1796.698	0
	OU	1797.452	0.754
tRNA Diversity vs. missense error rate	BM	-5956.54	140.5
	OU	-6097.04	0
GC vs. missense error rate	BM	-5948.735	118.309
	OU	-6067.044	0
GC vs. tRNA Diversity (GC-ending codons)	BM	1791.535	0
	OU	1793.672	2.137
GC vs. tRNA Diversity (AT-ending codons)	BM	653.397	77.831
	OU	575.566	0

Table S1. Model fit comparison of phylogenetic regressions based on either the Brownian Motion (BM) or Ornstein-Uhlenbeck (OU) models of trait evolution. Model comparisons are based on the Akaike Information Criterion (AIC). Models with the lower AIC are considered the better model.

Parameter	Value	P-Value
α_{Meso}(intercept)	49.0393	< 0.0001
$\beta_{Psychro}$	3.9119	0.3373
β_{Thermo}	-2.9006	0.15
β_{GC}	3.9649	0.5604

Table S2. Phylogenetic regression parameter estimates when comparing total tGCN across mesophiles, thermophiles, and psychrophiles while taking GC% into account. This can be represented by the formula $Total\ tGCN\ = \alpha_{Meso} + \beta_{Thermo}x_{Thermo} + \beta_{Psychro}x_{Psychro} + \beta_{GC}x_{GC}$, where x_{Thermo} and $x_{Psycrho}$ indicate if the bacteria is a thermophile or psychrophile (i.e., $x_{Thermo/Psychro} = 1$, and 0 otherwise). This means the slope estimates β_{Thermo} and $\beta_{Psychro}$represent the mean value of the total tGCN relative to a mesophilic bacteria. This regression assumed an OU model of trait evolution, which was 53 AIC units better than the same regression based on a BM model.

Parameter	Value	P-Value
α_{Meso}(intercept)	0.0018	< 0.0001
$\beta_{Psychro}$	-0.0000275	0.63090
β_{Thermo}	0.0000547	0.01164
β_{GC}	0.00089	< 0.0001

Table S3. Phylogenetic regression parameter estimates when comparing the median missense error rates across mesophiles, thermophiles, and psychrophiles while taking GC% into account. We note this This can be represented by the formula $Median\ Missense\ Error\ Rate = \alpha_{Meso} + \beta_{Thermo}x_{Thermo} + \beta_{Psychro}x_{Psychro} + \beta_{GC}x_{GC}$, where x_{Thermo} and $x_{Psycrho}$ indicate if the bacteria is a thermophile or psychrophile (i.e., $x_{Thermo/Psychro} = 1$, and 0 otherwise). This means the slope estimates β_{Thermo} and $\beta_{Psychro}$represent the mean value of the median missense error rates relative to a mesophilic bacteria. This regression assumed an OU model of trait evolution, which was 124 AIC units better than the same regression based on a BM model.
